# Lifestyle Intervention Using an Internet-Based Curriculum with Cell Phone Reminders for Obese Chinese Teens: A Randomized Controlled Study

**DOI:** 10.1371/journal.pone.0125673

**Published:** 2015-05-06

**Authors:** Anisha A. Abraham, Wing-Chi Chow, Hung-Kwan So, Benjamin Hon-Kei Yip, Albert M. Li, Shekhar M. Kumta, Jean Woo, Suk-Mei Chan, Esther Yuet-Ying Lau, E. Anthony S. Nelson

**Affiliations:** 1 Division of Family Medicine and Primary Health Care, The Jockey Club School of Public Health and Primary Care, The Chinese University of Hong Kong, Hong Kong SAR, China; 2 Department of Paediatrics, The Chinese University of Hong Kong, Hong Kong SAR, China; 3 Department of Orthopaedics & Traumatology, The Chinese University of Hong Kong, Hong Kong SAR, China; 4 Department of Medicine & Therapeutics, The Chinese University of Hong Kong, Hong Kong SAR, China; 5 Department of Psychology, The University of Hong Kong, Hong Kong SAR, China; Weill Cornell Medical College Qatar, QATAR

## Abstract

**Objectives:**

Obesity is an increasing public health problem affecting young people. The causes of obesity are multi-factorial among Chinese youth including lack of physical activity and poor eating habits. The use of an internet curriculum and cell phone reminders and texting may be an innovative means of increasing follow up and compliance with obese teens. The objectives of this study were to determine the feasibility of using an adapted internet curriculum and existing nutritional program along with cell phone follow up for obese Chinese teens.

**Design and Methods:**

This was a randomized controlled study involving obese teens receiving care at a paediatric obesity clinic of a tertiary care hospital in Hong Kong. Forty-eight subjects aged 12 to 18 years were randomized into three groups. The control group received usual care visits with a physician in the obesity clinic every three months. The first intervention (IT) group received usual care visits every three months plus a 12-week internet-based curriculum with cell phone calls/texts reminders. The second intervention group received usual care visits every three months plus four nutritional counselling sessions.

**Results:**

The use of the internet-based curriculum was shown to be feasible as evidenced by the high recruitment rate, internet log-in rate, compliance with completing the curriculum and responses to phone reminders. No significant differences in weight were found between IT, sLMP and control groups.

**Conclusion:**

An internet-based curriculum with cell phone reminders as a supplement to usual care of obesity is feasible. Further study is required to determine whether an internet plus text intervention can be both an effective and a cost-effective adjunct to changing weight in obese youth.

**Trial Registration:**

Chinese Clinical Trial Registry ChiCTR-TRC-12002624

## Introduction

Obesity is an increasing public health problem affecting young people. Approximately 16% of Hong Kong children and youth aged 6 to 18 years are overweight or obese [1]. The causes of obesity in children are multi-factorial and include lack of physical activity and poor eating habits. Behavioural treatment is the mainstay of weight-control programs for obese teens. Traditional behavioural-based programs are usually done face-to-face by health professionals and include strategies to increase physical activity and modify dietary habits [2]. However seeing medical professionals for one-to-one consultations to discuss weight management on a regular basis can be expensive and time-consuming.

According to a recent study commissioned by the Leisure and Cultural Services Department on the physical fitness of the Hong Kong population, less than 10% of young adolescents aged 13–19 years met the recommendation by the World Health Organization for accumulating 60 minutes of moderate-level-or-above intensity physical activity [3]. Many young people in Hong Kong do not have enough physical exercise on a regular basis due to the result-oriented educational system. In fact, pressure to succeed in school is very high in Hong Kong. In addition, there is significant competition for admission to universities. According to statistics from the University Grants Committee of Hong Kong and the Hong Kong Examinations and Assessment Authority, 29% of 114,646 candidates attained university places in 2012/2013 [4–6]. Examination pressure may thus lead to stress and mental health problems [7] and also pressure by parents and teachers on students to prioritise study over physical activity.

An eight week randomized controlled trial study called Web-Based Active Balance Childhood was conducted in the US among Chinese American adolescents (n = 63), aged 12 to 15, and their parents, in order to improve the teens’ dietary intake and physical activity [2]. The study was based on Transtheoretical Model-Stages of Change and social cognitive theory. Participants in the intervention group received their individually tailored curriculum based on their behavioural stage of change. The results were shown to be feasible and effective in improving general health, decreasing diastolic blood pressure and waist-to-hip ratio while increasing vegetable and fruit intake, the level of physical activity, and knowledge about both.

A Lifestyle Modification Programme (LMP) has been developed by the Centre for Nutritional Studies (CNS) at the Chinese University of Hong Kong [8]. It is a dietician/nutritionist-led intervention which aims at improving knowledge regarding diet and exercise and changing long term lifestyle. The program uses a patient-centred approach and cognitive behavioural concepts and provides individualized diet, physical activity and behavioural weight control plans for weight loss and long term weight maintenance [8]. The program is effective in weight management in Chinese obese adults, both with and without diabetes, [9] and in normalizing liver fat in patients with non-alcoholic fatty liver disease [10]. However the LMP is resource intensive consisting of weekly counselling during the first 3 months, plus advice from an exercise instructor.

Recent studies have shown internet-delivered interventions can be an effective means for acquiring knowledge, for improving health behaviours and for general health maintenance in adults [11,12]. In addition to web-based programs, cell phone texting and reminders may be an innovative means of increasing follow-up and compliance with obese youth [13,14]. It also enhances care in other chronic disease such as diabetes. A randomized control trial study involving a text messaging system was conducted among paediatric patients with Type 1 diabetes (n = 92), aged 8 to18 years [15]. Results showed that subjects receiving intensive insulin therapy with daily personalized text messages had an improvement in HbA_1c_ compared with those on conventional therapy with or without daily personalized text messages. The study also demonstrated that daily personalized text messages were associated with improvements in diabetes self-efficacy and self-reported adherence. Finally, a systematic review of 26 studies involving internet-based health behaviour change interventions targeting adolescents or young adults showed that a combination of tailored communication and use of reminders and incentives were largely effective [14].

Involving parents in obesity management programmes is likely important as evidenced by an 18- month randomized controlled trial involving family-based and behavioural intervention in 192 obese American children aged 8 to 12 years [16]. Families and children in the intervention group were provided 20 educational group sessions (60 minutes each) for six months. The topics discussed included providing behavioural modification techniques (self-monitoring, stimulus control, and stepwise goal-setting), promoting body image, controlling emotional eating, parental modelling, increasing physical activity and decreasing sedentary behaviours. A further six booster sessions (3 group sessions and 3 telephone calls) were provided for an additional six months. Families and children in the control group were given two nutrition consultation sessions during month 0 to month 6. No additional contact was provided between assessments from month 6 to month 18. The results showed a significant decrease in the percentage of children who were overweight at 6 months in the intervention group compared with the control group. Also compared with the control group, children in the intervention group had significant improvements in waist circumference, systolic blood pressure, percent body fat and total body fat. A number of other studies have also shown that parental involvement can play an important role in weight management in obese children [2,17–19]. As Chinese parents are typically involved in many aspects of their children’s health, it is important to add family-based components to childhood-obesity prevention programs.

To date there have been no reports of the use of internet-based obesity prevention programmes with cell phone reminders in Hong Kong adolescents. The purpose of this pilot study was to assess the feasibility of using an adapted internet-based curriculum with cell phone follow-up and an existing nutritional program as adjuncts to usual care for obese adolescents and their parents.

## Methods and Procedures

The protocol for this trial and supporting CONSORT checklist are available as supporting information; see S1 CONSORT Checklist and S1 Protocol.

The study was conducted in two phases. The first phase included curriculum development and focus group discussions among obese adolescents attending the Obesity Clinic at a tertiary care hospital in Hong Kong. The second phase was a randomized controlled study involving 3 arms (Control Group vs Simplified Lifestyle Modification Programme Group (simplified LMP) vs Internet Group (IT)).

### Phase 1: Curriculum development and preliminary focus group discussions

The curriculum was adapted and modified from an existing ABC curriculum validated for use in Chinese American adolescents in an urban community setting [2]. The modified internet curriculum consisted of twelve 15-minute interactive sessions consisting of reflective questions, wrap up quizzes and matching games. Information related to nutrition such as the Food Pyramid, portion size, types of rice consumption (brown rice vs white rice), the importance of increasing energy expenditure through physical activity, such as brisk walking and decreasing screen time, were discussed. Relaxation mindful eating practices were also included for stress management (Table 1).

**Table 1:** 12-week internet based curriculum.

Lesson	Intervention
1	Introduction-Basic definition and welcome to internet curriculum
2	Obesity Causes and Consequences- understand the main reasons for increasing weight and what effects this can have on your health
3	Nutrition Basics-Food Pyramid-understand food and health
4	Nutrition Basics- learn about portion size and how to make smart good choices
5	Eating healthy outside of the home
6	Physical Activity-Understand the importance of an adequate activity level
7	Physical Activity-Being cool and active: various fun activities for youth and families
8	Physical Activity-Being yourself and using fun ways to improve your health and maintain a healthy lifestyle
9	TV/computer time-Learn alternatives to watching TV and using the computer
10	Stress/ coping- Understand how the body works and how to recognize and cope with feelings
11	Stress/coping-Using various relaxation techniques and develop healthy coping
12	Final Summary- Wrap up all the curriculum

To test the curriculum, five focus group discussions were conducted among obese teens (aged 12 to 18 years) attending the Obesity Clinic. To maintain confidentiality, all subjects were assigned a moniker in place of their real name for the session. All subjects were asked to review the curriculum on laptops independently for 35 minutes. Then, participants were asked open-ended questions about the curriculum, the use of social media and texting in the study. All sessions were conducted in Cantonese and lasted around 90 minutes, including a 10 minute de-briefing at the end. All conversations were recorded and transcribed. Transcripts were reviewed twice to check accuracy and to gain an overview of transcription. Data were coded using open and axial coding and categorized into broad themes. The internet curriculum was further refined based on the results and feedback of the focus groups.

### Phase 2: Randomized Controlled Study

#### (1) Research subjects

Patients were recruited from the Obesity Clinic at a tertiary care hospital in Hong Kong by a research assistant. Inclusion criteria were: (i) being aged 12 to 18 years; (ii) having a BMI greater than 95^th^ percentile for age; (iii) attending the Paediatric Obesity and Lipid Clinic; and (iv) being proficient in Cantonese. Exclusion criteria were: (i) BMI below 95^th^ percentile of local reference; (ii) concurrent participation in any clinical trial or dietary intervention program; or a severe medical illness. Written informed consent/assent was obtained from parents and the teen subjects.

#### (2) Sample size

Convenience sampling was employed for this pilot feasibility study with a sample size of 48, consisting of 16 subjects in the control group and 16 subjects in each of the two different intervention groups.

#### (3) Design and randomization

After obtaining informed consent, subjects were randomly assigned to either one of two intervention groups or the control group. To ensure balanced allocation, the randomization sequence was computer-generated in variable blocks of six. A paediatrician assigned subjects to interventions according to the randomization assignment. A research assistant, who was blinded to randomization assignment, oversaw recruitment, enrolment, and follow up. The entire study period was 24 weeks.

(3i) Control group

The control group received usual care visits with a physician at the Obesity Clinic at baseline/week 0 (T0), mid-point/week 12 (T12) and at the end of the study/week 24 (T24). Usual care consisted of a focused dietary and physical activity history, medical history, physical examination, laboratory screening and obesity counselling.

(3ii) Internet (IT) intervention group

The subjects in the internet intervention (IT) group also received usual care visits to the obesity clinic at baseline (T0), midpoint (T12) and at the end of the study (T24), together with the 12-week (from baseline to week 12) internet-based curriculum (Table 1) and cell phone follow-up over 6 months. Subjects were asked to set specific goals related to (a) diet and (b) physical activity at baseline and then every month. Examples included “I will try to consume 5 servings of fruit and veggies every day or I will try to walk 30 minutes per day”. The research assistant sent weekly semi-personalized SMS (short message service) messages. These messages incorporated subjects’ diet and exercise goals to participants’ phones throughout the study. Subjects were asked to reply with an emotion icon to represent whether they had achieved their targets during the week.

(3iii) Simplified Lifestyle Modification Programme (sLMP) intervention group

The LMP is a dietician/nutritionist-led intervention using a patient-centred approach and cognitive behavioural concepts to improve knowledge regarding diet and exercise as well as changing long term lifestyle. The second intervention group received usual care visits with a physician at the Obesity Clinic at baseline (T0), midpoint (T12) and at the end of the study (T24) and a simplified version of the LMP consisting of four meetings with a nutritionist over three months. The simplified LMP (sLMP) was used in this pilot study because of limited budget and resources. The number of counselling sessions was reduced from 12 sessions in the original LMP to 4 sessions. The sessions were conducted at baseline (T0), 2 weeks (T2), 4 weeks (T4) and 12 weeks (T12). These nutritional counselling sessions were conducted by a nutritionist familiar with the LMP.

During the initial one-hour nutritional assessment, the parent-teen pair completed a behavioural assessment questionnaire and the nutritionist obtained information on the subject’s medical history, anthropometric measurements, current eating and lifestyle patterns and readiness to change. The nutritionist provided personalized dietary and exercise advice at each visit. The nutritionist also encouraged a varied balanced diet with an emphasis on the American Dietetic Association guidelines with low-fat, low-glycaemic index and low calorie products in appropriate portions.

#### (4)Study outcomes

(4.1)Primary outcome

The primary outcome of the study was the feasibility of adapting and using an existing internet curriculum and nutritional program along with cell phone follow up for obese teens. This was assessed by examining recruitment and retention rate, internet log-in rate, compliance and satisfaction with counselling, curriculum and cell phone communication.

(4.2)Secondary outcomes

The secondary outcomes of the study were subjects’ physical activity level, dietary intake, stress level, and knowledge related to nutrition/physical activity, as well as weight and blood pressure. These indices were also used to determine the effectiveness of the program. All subjects completed anthropometric measurements and self-administered questionnaires regarding their physical activity [20], depression, anxiety and stress levels (DASS-21) [21] and dietary knowledge [22] at baseline (T0), mid-point (T12) and at the end of the study (T24). Anthropometric measurements included standing height, waist and hip circumferences, body weight and blood pressure.

#### (5)Statistical analysis

Descriptive statistics were calculated for demographic characteristics, recruitment and retention rate. Average log-in rate and compliance with curriculum rate were computed as a percentage of sessions the subject logged on and completed activities over the 12 sessions. Compliance with the curriculum and cell phone communication rates were computed as percentages. Continuous and categorical data were presented as medians (interquartile range (IQR)) and number (percentage). The quantitative variables among the three groups were compared using Kruskal—Wallis test and post hoc analysis was carried out using Mann—Whitney test with Bonferroni corrected significance level of 1.7% set for follow-up comparisons. Repeated measures analysis was conducted with function of time and group effects to examine the changes over times within-groups and between groups. Satisfaction scores with counselling, curriculum and cell phone communication were presented as non-parametric paired comparisons using the Wilcoxon signed rank test. A p-value < 0.05 was considered statistically significant. SPSS for Windows software (version 18.0, SPSS Inc., Chicago, IL, USA) was used for analysis.

#### (6) Ethical approval

The study was conducted in compliance with the ICH-GCP and Declaration of Helsinki and was approved by the Joint Clinical Research Ethics Committee of the Chinese University of Hong Kong and the New Territories East Cluster [CRE-2011.619-T]. The trial was registered with the Chinese Clinical Trial Registry (ChiCTR-TRC-12002624).Written informed consent/assent was obtained from parents and teen subjects.

## Results

### Phase 1: Focus groups

A total of 11 obese Chinese teens (10 males and 1 female) were recruited. The broad themes identified included barriers to losing weight, means of improving the medical visit and the acceptability of the cell phone follow-up and support with weekly text message reminders. Most respondents expressed that time restrictions and a heavy school workload were major barriers to reducing weight. Participants also felt that the use of web-based curriculum in addition to an individual medical consultation with a paediatrician was useful. Respondents indicated that the web-based curriculum could be used as a tool to manage their weight and perhaps prevent further weight gain by controlling their food intake and increasing their amount of physical activity. Participants also agreed that weekly individualized text message reminders could be a way to enhance their motivation to adopt healthy behaviours.

The internet curriculum was further refined on the basis of the feedback from the focus groups. The limitation of conducting focus groups was that it was challenging to recruit female participants as four of the recruited female participants dropped out before group sessions because they wanted to spend more time preparing for exams.

### Phase 2: Randomized Controlled Study

#### (1) Subject Demographics

**Fig 1:**
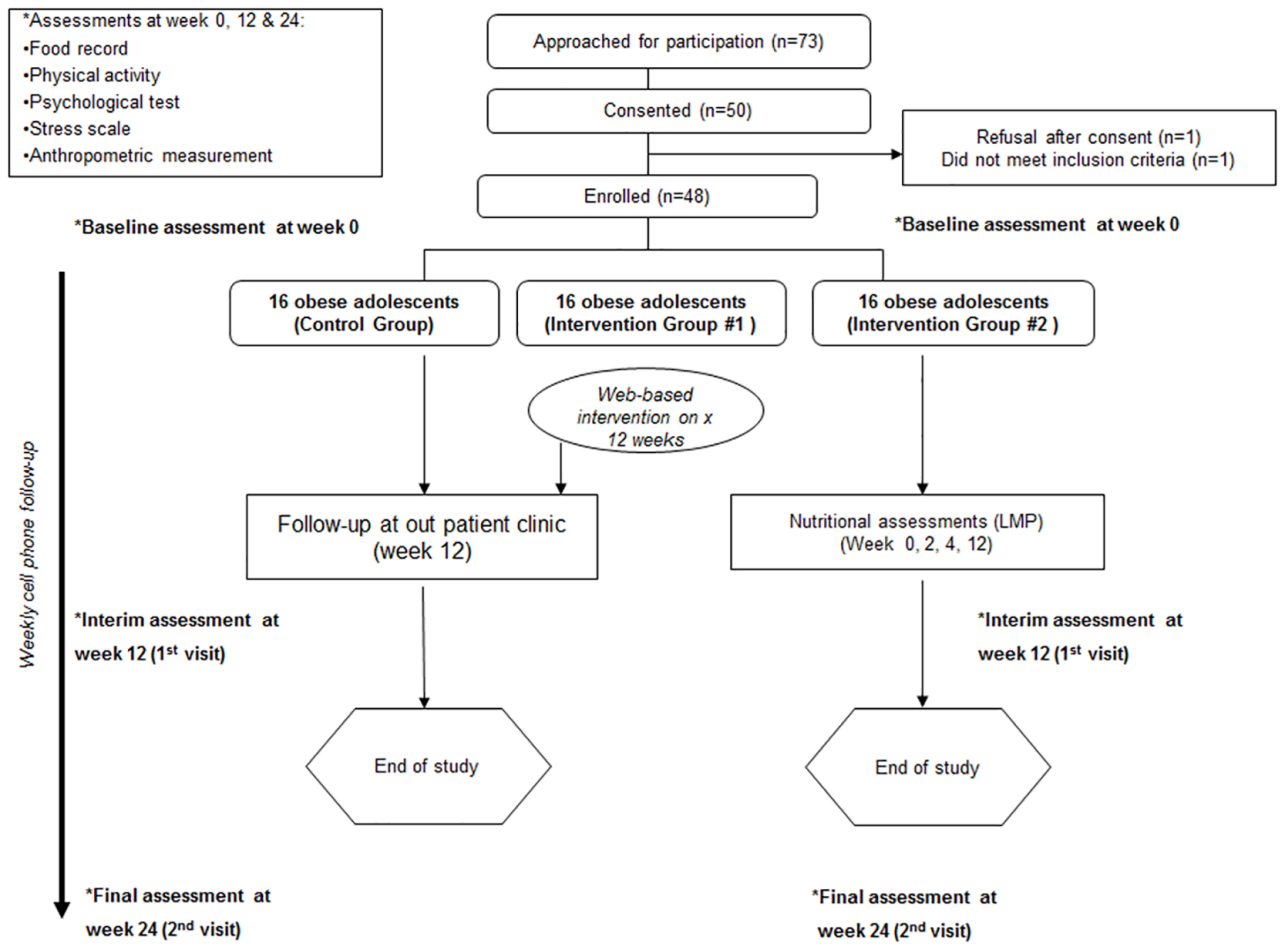
Consort table.

**Fig 2:**
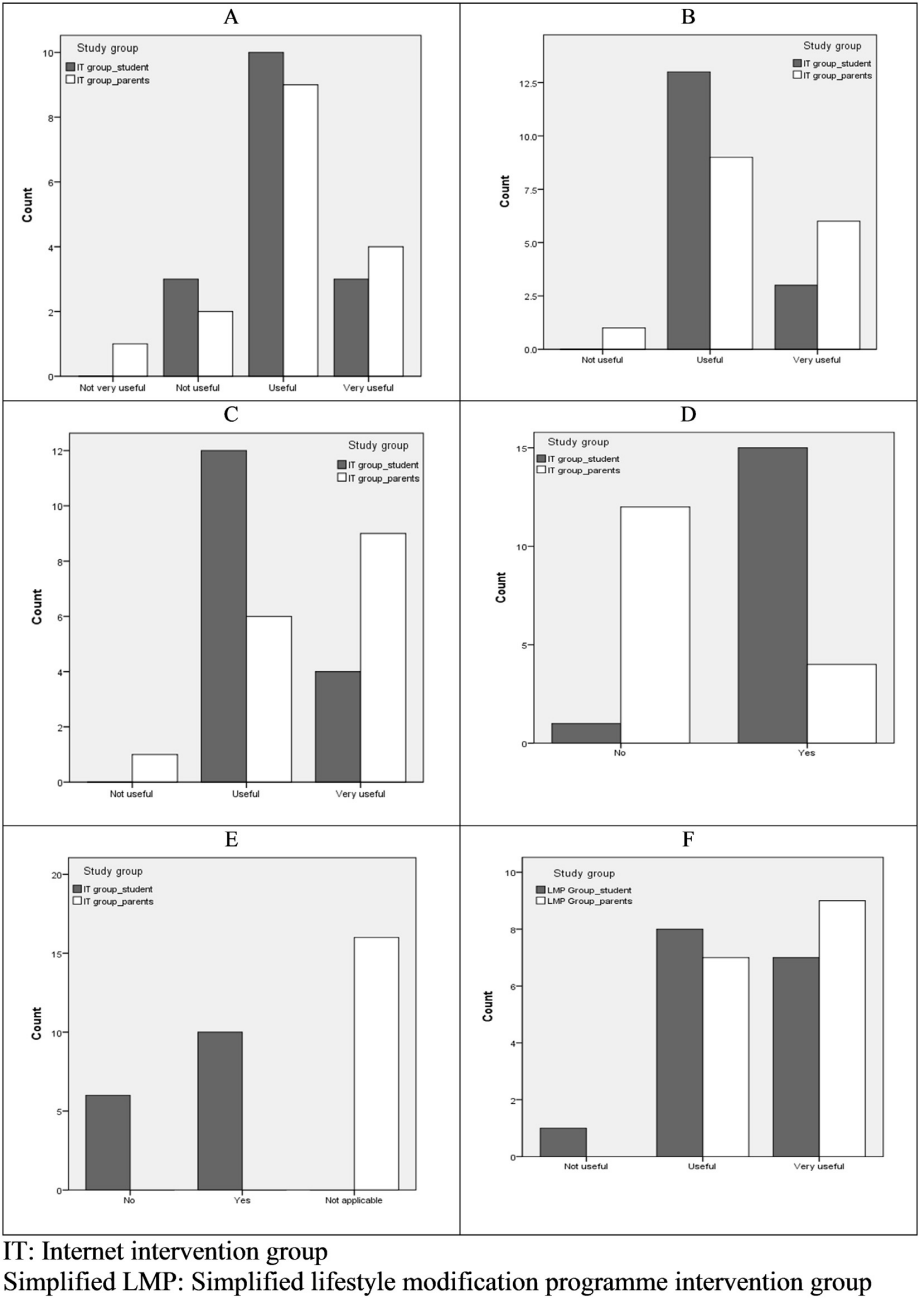
Participation rates and satisfaction levels of intervention subjects at final visit (week 24). Satisfaction levels of subjects in the internet intervention group (IT) regarding the internet curriculum (A), cell phone communication (B), goal setting (C) and IT subjects’ log-in (D) and curriculum completion rates (E) for online curriculum at final visit (week 24). Satisfaction levels of subjects in simplified lifestyle modification programme (sLMP) group with nutritional counselling at final visit (F).

From January 2013 to early July 2013, 73 obese subjects aged 12 to 18 years were invited to join the study. Of these subjects, 50 (68%) agreed to participate. Two subjects were later excluded, one subject refused to participate after week 2 and one subject was found to be too young to take part in the study (Fig 1). There were no significant differences among the 48 subjects in the three groups with respect to their demographic data (Table 2). The mean age was 14.4 years, with a median body weight of 84.9 kg (77.7–91.5). There were 29 (60.4%) males and the ratio of obese boys to girls was consistent with previous Hong Kong studies involving obese youth [1]. Table 3 and Fig 2 summarize the satisfaction levels with counselling, curriculum and cell phone communication in the intervention groups.

**Table 2:** Demographic characteristics of the control group, internet (IT) intervention group and simplified Lifestyle Modification Programme (sLMP) group.

	Control group (n = 16)	IT group (n = 16)	sLMP group (n = 16)
Male (n/%)	10 (62)	9 (56)	10 (62)
Age (median, IQR)	14.3 (13.5–15.8)	14.9 (13.7–16.2)	14.1 (13.5–15.3)
*Peternal education level*:			
Primary (n/%)	2 (13)	3 (19)	2 (13)
Secondary (n/%)	10 (62)	12 (75)	10 (62)
Tertiary (n/%)	4 (25)	1 (6)	4 (25)
*Maternal education level*:			
Primary (n/%)	2 (13)	1 (6)	3 (19)
Secondary (n/%)	13 (81)	14 (88)	11 (68)
Tertiary (n/%)	1 (6)	1 (6)	2 (13)
*Family monthly income*:			
HKD10,000–20,000 (n/%)	8 (50)	9 (56)	6 (38)
HKD20,001–30,000 (n/%)	5 (31)	2 (13)	5 (31)
≥HKD30,001 (n/%)	3 (19)	5 (31)	5 (31)
Paternal BMI (median, IQR)	25.8 (22.7–29.2)	24.3 (22.9–29.6)	26.0(24.2–30.2)
Maternal BMI (median, IQR)	26.4 (24.0–30.2)	25.1 (21.4–27.1)	23.9 (20.9–29.5)
Paternal obesity	9 (56)	7 (44)	11 (68)
Maternal obesity	10 (62)	8 (50)	7 (44)

IQR: interquartile range. Maternal and paternal obesity defined as BMI> = 25

**Table 3:** Summary of satisfaction levels with usual care, nutrition counselling, curriculum and cell phone communication from the control, internet (IT) and simplified lifestyle modification programme (sLMP) groups completed at final visit (week 24).

		Control group		IT group		sLMP group	
Questions		Students, (n = 16)	Parents, (n = 16)	Students, (n = 16)	Parents, (n = 16)	Students, (n = 16)	Parents, (n = 16)
How useful were the consultations with a health care provider to manage your (child's) obesity?	Not very useful	0	0	0	0	0	0
	Not useful	6.2	0	12.5	6.2	0	6.2
	Useful	81.3	25	68.8	56.3	56.3	43.8
	Very useful	12.5	75	18.7	37.5	43.7	50
Do you think that the follow up appointments should occur more often than every 3 months?	Strongly disagree	0	0	6.2	0	0	6.2
	Disagree	18.7	0	25	12.5	6.2	12.5
	Agree	62.6	50	68.8	50	62.5	37.5
	Strongly agree	18.7	50	0	37.5	31.3	43.8
How useful was the curriculum in terms of managing your (child's) obesity?	Not very useful	No consultation and curriculum intervention		0	6.2	No curriculum intervention	
	Not useful			18.7	12.5		
	Useful			62.6	56.3		
	Very useful			18.7	25		
How useful was the weekly SMS in terms of managing your (child's) obesity?	Not very useful			0	0		
	Not useful			0	6.2		
	Useful			81.3	56.3		
	Very useful			18.7	37.5		
How useful were setting PA and Dietary goals in terms of managing your (child's) obesity?	Not very useful			0	0		
	Not useful			0	6.2		
	Useful			75	37.5		
	Very useful			25	56.3		
Have you logged into the Moodle?	Yes			100	25		
	No			0	75		
Have you completed all lessons?	Yes			62.5	Not applicable		
	No			37.5			
How useful was the consultation with the nutritionist in terms of managing your (child's) obesity?	Not very useful			No consultation intervention		0	0
	Not useful					6.2	0
	Useful					50	43.7
	Very useful					43.8	56.3

Values are expressed as column percentages

#### (2) Control Group Results

All 16 recruited subjects completed visits at baseline (T0), mid-point (T12) and at the end of study (T24). All parents and the majority of subjects (93.5%) found the consultations with a paediatrician useful in terms of managing obesity (Table 3).

#### (3) IT Intervention(IT) Group Results

All 16 recruited subjects completed visits at baseline (T0), mid-point (T12) and at the end of study (T24).

(3i) IT Group Curriculum Participation

All subjects logged in to the system but only 14 out of 16 subjects read the curriculum (87.5%). Curriculum review was determined by participants’ score on a quiz at the end of the weekly lesson. Among the 14 subjects, the majority (71%) completed all lessons. Only four parents read the curriculum.

(3ii) IT Group Text Message Usage

**Fig 3:**
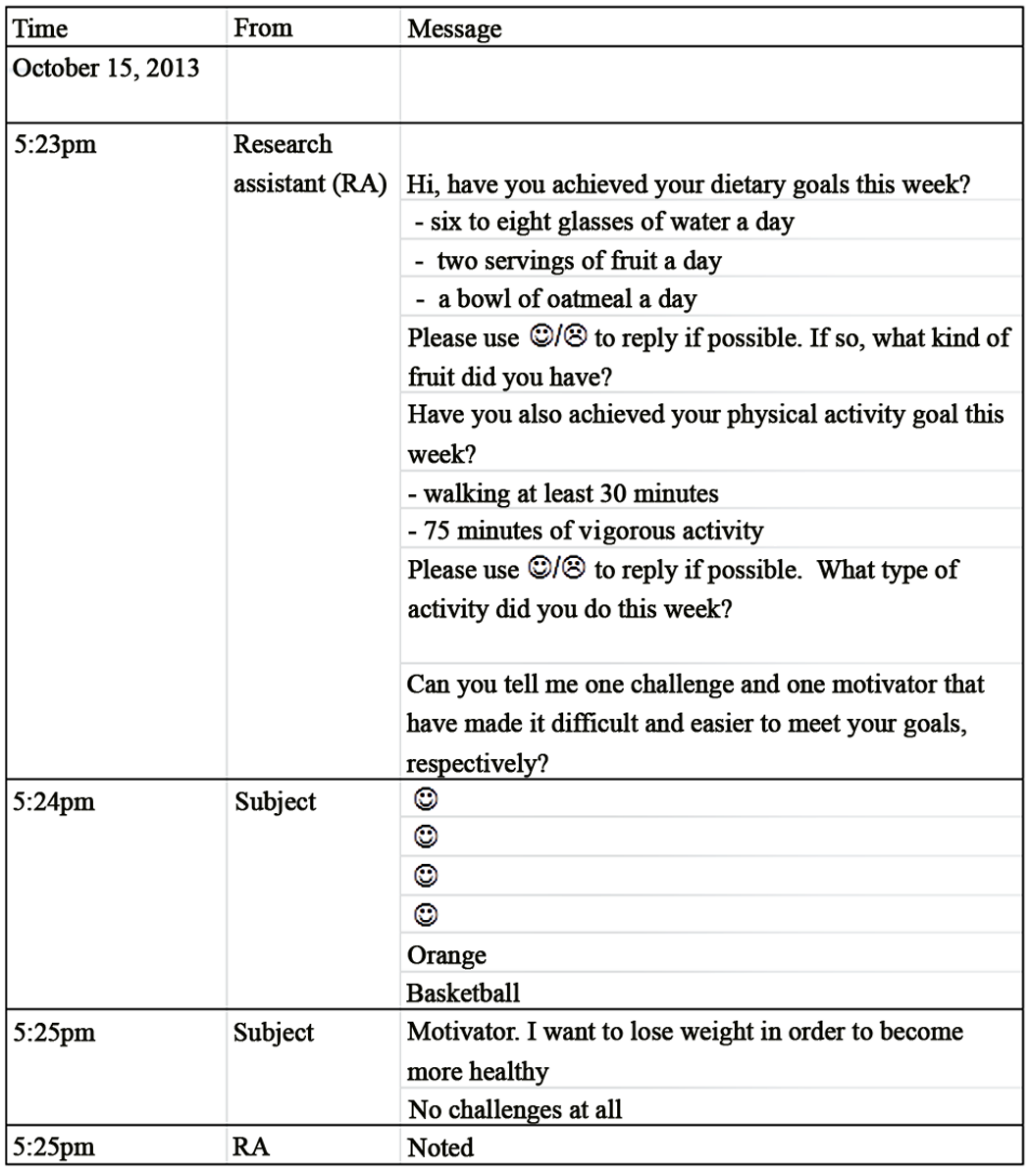
Sample of a text message interaction with an intervention subject. A sample semi-personalized health message interaction log recording interactions between the research assistant and a subject in the internet (IT) intervention group.

A total of 400 messages were sent to 16 subjects. 15 subjects opted to receive the message by Whatsapp (a communication application on smartphones) via cell phone and one preferred email only. The response rate to dietary goals and exercise goals were 78.3% and 77.5%, respectively. Fig 3 shows a sample of the semi-personalized health message log recording the interactions between the research assistant and subjects. The research assistant spent an average of 2 hours per week to send out the personalized cell phone reminders to the 16 participants.

(3iii) IT Group Satisfaction Level

The majority of subjects and parents found the consultations with a paediatrician useful or very useful in terms of managing obesity (Table 3). Satisfaction with the curriculum, goals setting and cell phone reminders was also high (Table 3 and Fig 2A–2C).

#### (4) Simplified LMP (sLMP) Intervention Group Results

(4i) Nutritional Counselling Participation

All 16 recruited subjects in the sLMP group completed three visits at baseline (T0), mid-point (T12) and at the end of study (T24). The majority of the subjects (94%) also completed all nutritional counselling sessions. However, the percentages of participants that showed up on time for the nutritional counselling varied. They were 100% (T0), 81.3% (T2), 62.5% (T4) and 0% (T12), respectively. Subjects who failed to show up on time cited difficult school schedules, heavy workload and laziness as the main reasons for their late arrival.

(4ii) sLMP Satisfaction with Nutritional Counselling

The majority of students and parents found the consultations with a paediatrician useful or very useful in terms of managing obesity (Table 3). All parents and 94% of subjects found the counselling with the nutritionist useful or very useful in terms of managing obesity (Table 3 and Fig 2F).

#### (5) Secondary outcome measures

**Fig 4:**
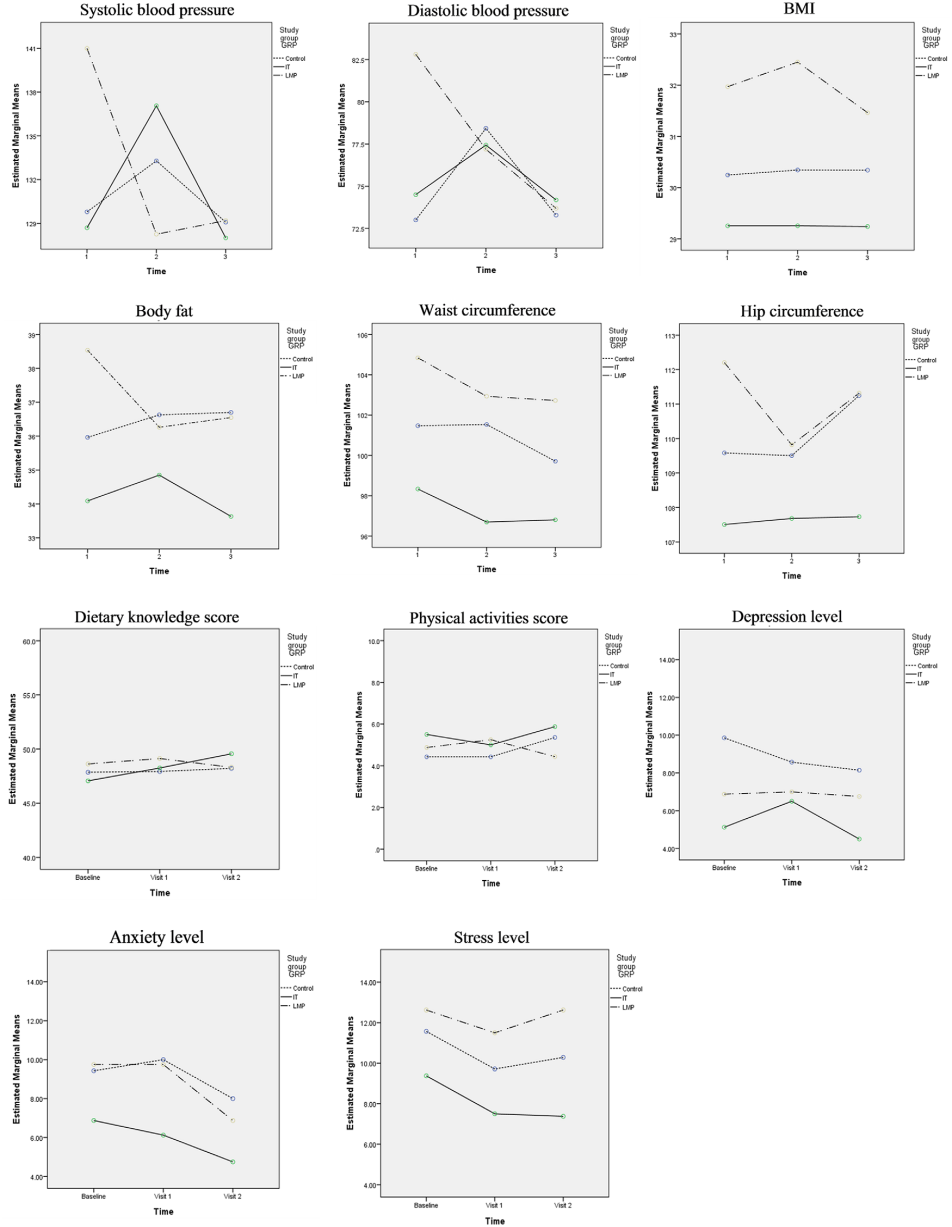
Comparison of secondary outcomes between subject groups at each visit. Comparison (repeated measures) between control (usual-care) group, internet (IT) intervention group and simplified lifestyle modification programme (sLMP) group for blood pressure, anthropometric measures, and scores for dietary knowledge, physical activity, depression, stress and anxiety over three time periods: baseline, 1^st^ visit (week 12) and final visit (week 24).

The secondary outcome measures of the study were BP, body fat, BMI, physical activity, stress (DASS 21) and dietary knowledge scores at T0, T12 and T24. A borderline significant decrease in systolic (p = .046) and diastolic (p = .047) pressure was noted at T12 and T24 compared to T0 in the sLMP group. No other consistent significant differences were shown in these parameters or scores between the groups or within groups over the course of the study (Table 4 and Fig 4). Using the BMI as the outcome of interest, the post hoc calculation of the calibrated observed power was determined to be 0.122. This is based on the mean difference of change of BMI between the adapted intern-based curriculum (IT) intervention and control group (-3.32, with a pooled SD of 1.13). The effect size is 0.285 and is equivalent to a moderate Cohen’s d. Of note, given this is the true effect size, a future RCT with a sample size of 388 (194 in the IT group and 194 in the control group) would achieve 80% power to reject the null hypothesis.

**Table 4:** Comparison of anthropometric measures, dietary knowledge score, physical activity score and Depression, Anxiety and Stress Scale (DASS) score between the control, internet (IT) and simplified lifestyle modification programme (sLMP) groups at baseline, 1^st^ visit (week 12) and final visit (week 24).

	Baseline				1st visit				2nd visit			
	Control group (n = 16)	IT group (n = 16)	sLMP group (n = 16)	*p*	Control group (n = 16)	IT group (n = 16)	sLMP group (n = 16)	*p*	Control group (n = 16)	IT group (n = 16)	sLMP group (n = 16)	*p*
*SBP (mmHg)	131 (118–143)	123 (118–135)	138 (129–150)	.015	132 (126–142)	138 (127–145)	130 (125–134)	0.038	131 (121–139)	129 (122–136)	130 (121–137)	0.88
*DBP (mmHg)	73 (65–83)	74 (68–82)	83 (74–87)	.045	80 (75–82)	77 (71–83)	77 (71–82)	0.925	76 (64–82)	75 (67–80)	74 (67–83)	1
BMI	30.1 (28.4–32.3)	29.3 (26.7–30.9)	31.5 (29.8–33.7)	.032	30.3 (28.5–31.9)	28.6 (26.7–31.4)	30.9 (28.8–33.4)	0.142	30.5 (28.7–32.0)	28.4 (26.7–31.9)	31.0 (39.6–34.1)	0.065
Body fat (%)	36.5 (31.3–41.0)	33.6 (27.3–42.0)	38.1 (31.6–44.9)	0.258	36.2 (31.1–41.8)	33.0 (27.3–40.3)	36.9 (33.3–40.9)	0.418	37.6 (30.5–43.6)	31.6 (26.0–42.4)	37.3 (29.8–42.0)	0.274
WC (cm)	103.0 (96.1–108.7)	96.6 (92.2–106.7)	101.8 (97.5–111.3)	0.073	102.8 (96.6–109.3)	96.4 (90.6–101.5)	99.0 (94.3–113.9)	0.220	101.6 (93.5–107.9)	94.9 (92.0–100.5)	100.5 (96.1–108.0)	0.065
HC (cm)	110.1 (107.5–114.8)	106.6 (105.6–109.9)	111.0 (106.1–117.6)	0.070	109.1 (107.9–113.2)	107.3 (104.8–110.0)	107.3 (105.0–115.9)	0.497	111.5 (106.3–120.0)	107.9 (103.3–111.8)	110.5 (106.7–115.5)	0.118
Dietary knowledge score (max. score: 75)	48.0 (47.0–49.0)	48.0 (46.0–49.0)	51.0 (48.0–52.0)	0.204	46.0 (45.0–50.0)	50.0 (46.0–52.0)	51.0 (43.0–54.0)	0.884	47.5 (44.5–49.5)	48.5 (47.0–50.5)	47.5 (44.0–54.0)	0.363
Physical activity score (max.score:10)	4.5 (2.0–6.5)	6.0 (3.5–6.5)	4.0 (3.0–7.0)	0.566	5.0 (2.0–7.0)	5.0 (3.0–6.0)	5.5 (3.5–7.0)	0.675	6.0 (3.0–6.5)	6.0 (4.5–7.0)	4.0(3.0–6.0)	0.062
**DASS**												
Depression level(max. score: 42)	8.0 (2.0–14.0)	3.0 (1.0–6.0)	7.0 (3.0–11.0)	0.219	9.0 (2.0–14.0)	4.0 (0–12.0)	6.0 (1.0–11.0)	0.578	6.0 (1.0–13.0)	1.0 (0–8.0)	5.0 (2.0–10.0)	0.295
Anxiety level (max. score: 42)	8.0 (6.0–12.0)	4.0 (2.0–9.0)	10.0 (4.0–12.0)	0.139	10.0 (4.0–16.0)	3.0 (0–12.0)	8.0 (5.0–16.0)	0.165	6.0 (3.0–11.0)	1.0 (0–7.0)	8.0 (2.0–10.0)	0.211
Stress level (max. score: 42)	10.0 (5.0–15.0)	8.0 (2.0–14.0)	10.0 (8.0–16.0)	0.343	11.0 (1.0–18.0)	3.0 (1.0–14.0)	12.0 (4.0–19.0)	0.371	9.0 (2.0–18.0)	3.0 (2.0–12.0)	12.0 (6.0–18.0)	0.218

Values are expressed as median and interquartile range. SBP: Systolic blood pressure. DBP: Diastolic blood pressure. BMI: Body mass index. WC: Waist circumference. HC: Hip circumference. DASS: Depression, Anxiety and Stress Scale.

*SBP and DBP of sLMP group at 1^st^ visit and 2^nd^ visit were significantly lower than the baseline. Comparisons correspond to repeated ANOVA P<0.05

#### (6) Dietary intake

Subjects reported eating generally three meals per day. Many subjects skipped breakfast or ate white bread. Lunch and dinner mainly consisted of 1–2 bowls of steamed white rice, stir-fried meat such as chicken or beef and vegetables such as choy sum or bok choi. Of all subjects, 39.5% of them reported snacking on sweets, candy, cookies, biscuits or drinking carbonated beverages during the week. In addition, a few participants reported eating late at night or between meals.

In general, all participants’ diets were high in carbohydrates such as white rice with a high overall glycemic index. Participants were counselled to reduce glycemic index and switch to brown rice or whole grain bread. However, many participants found it difficult for their families to incorporate these changes.

## Discussion

### (1)Primary Objective

The primary outcome measure was to determine feasibility of an internet-based curriculum with cell phone follow-up for obese youth in Hong Kong. The study shows that the curriculum is feasible as evidenced by the relatively high recruitment rate, high retention rate, high internet log-in rate, and reasonable compliance with completion of online curriculum sessions and use of cell phone reminders. Patients also reported good satisfaction with the curriculum. The 2 hours per week required to send out the personalized cell phone reminders to the 16 participants highlights the need for additional manpower to provide individualised cell phone reminders in a busy hospital based setting. As such, an automated phone reminder system may be more time-effective and less costly, although it may be less tailored to individual patient needs [23].

Although over 90% of subjects in the sLMP attended all four nutrition counselling, most subjects did not bring their food records for counselling and most parents reported that they did not always follow the suggestions given by the nutritionist. Therefore, a phone or email reminder may be necessary to encourage patients to bring in food records. In general, all participants’ diets were predominately high in carbohydrates and had a high glycemic index. This is in comparison to the diets of obese Western youth which are also high in refined sweets and fat content. Subjects appeared to have difficulty following dietary advice given such reducing the overall glycemic index by switching to brown rice. Interestingly, nearly all parents and subjects rated the counselling with the nutritionist useful or very useful in terms of managing obesity.

Parents and subjects in both the IT intervention group and the sLMP group rated the interventions useful or very useful. Both intervention groups, and the control group, agreed that the follow-up appointments with a paediatrician should occur more frequently than every 3 months. Results of a recent meta-analysis also suggest that an in-person contact approach is superior to a technology-based approach from the viewpoint of the amount of weight loss, and if an internet program is used, it needs to include the component of a face-to-face program for participants to achieve weight loss [24]. Health behaviour change is a complex process and is influenced by a variety of physical, psychological, social and environmental factors. Further investigation of the interplay between these factors and weight loss outcomes is warranted to help determine the most appropriate frequency of health provider contacts for the management of obese teens.

### (2)Secondary Objective

The secondary objective was to examine the BP, body fat, BMI, physical activity, stress (DASS 21) and dietary knowledge scores. Although the study was not powered to show the effectiveness of the IT or sLMP interventions, no trends were apparent. The relatively small sample size of this pilot study could explain a lack of impact of any of these interventions on these outcomes. However it is also possible that the utility of these interventions is limited.

The original LMP has previously been shown to successfully reduce weight in both obese adolescents and adults. However the inability of the sLMP to show a consistent beneficial trend in the current study may reflect the modifications made. Compared to the original LMP, the frequency of counselling sessions were reduced in the sLMP. Moreover, the exercise component which is delivered by the exercise instructor in the original LMP was delivered by the nutritionist in this study. It is possible that both of these components could have affected the effectiveness of the sLMP. Lack of apparent effectiveness of our interventions could also be the result of the relatively infrequent contacts with the health care providers (every three months) as part of usual care. Since contact frequency has been shown to be an important factor for weight-loss success, a higher frequency of contact with a health care professional may be necessary to provide sufficient motivation and encouragement to follow lifestyle advice [25], including the additional advice contained in our interventions.

Previous studies have shown parental involvement is very important to achieve weight loss in children [2,16–19]. However, in this study parents were not significantly involved in the internet curriculum as evidenced by their low rate of signing in. Parents may not be using the computers as often as their children. It is possible that parents may also need frequent reminders to help their children to maintain goals. In some cases, students reported to their health care providers that their parents said that their academic performance was more important than engagement in physical activity and told them to stop physical activity during exam time. Parents need to be educated regarding the long term benefits of exercise for obesity even during school and exam time. Overall, incorporating a parent education program and actively involving parents should be an essential part of the internet-based curriculum program in the future.

### Limitations

Our subjects were mainly recruited at the end of the school year and the initial visits occurred mainly during the school holidays. However the final visits occurred after subjects had returned to school when work and exam pressures may have resumed. A potential limitation of our study is that feasibility of the interventions was affected by school holidays and exam times. It is also possible that the self-report measures used to determine feasibility of the interventions and subject satisfaction could lack reliability and validity.

## Conclusions

This is the first study to assess the feasibility of using both an internet-based curriculum with cell phone follow-up and a simplified LMP (sLMP), consisting of four nutritional counselling sessions, as adjuncts to usual care for obese adolescents and their parents. An internet-based curriculum as a supplement to usual care of childhood obesity is feasible, but further study is required to determine whether the use of an internet-based curriculum with/without cell phone reminders can be both an effective and a cost-effective adjunct to changing weight and promoting a healthy lifestyle in obese youth. More research is needed to determine how best to further incorporate technology such as the internet and cell phone reminders into the traditional labour intensive in-person approach.

## Supporting Information

S1 CONSORT Checklist (DOC)

S1 Protocol (PDF)
