# Supplementary material for: Lifestyle Intervention Using an Internet-Based Curriculum with Cell Phone Reminders for Obese Chinese Teens: A Randomized Controlled Study
Source: PLoS One. 2015 May 6;10(5):e0125673. doi: 10.1371/journal.pone.0125673 (PMC4422741; doi:10.1371/journal.pone.0125673)
Supplement: S1 Protocol — (PDF) [file pone.0125673.s002.pdf]

**Research Protocol**

**Study title: Lifestyle intervention using an internet-based curriculum with cell phone reminders among obese Chinese adolescents: A randomized controlled pilot study**

**Investigators:** Edmund Anthony NELSON, Anisha ABRAHAM, Albert M. LI, Tan CHOW, Jean WOO, Ruth Suk Mei CHAN, Esther LAU and Hung Kwan SO

**Compliance:** This study will be conducted in compliance with ICH-GCP and Declaration of Helsinki.

*Study design:* Randomized controlled trial.

*Inclusion criteria:* Adolescents aged 12 -18 years with primary obesity attending the Obesity and Lipid Clinic at the Prince of Wales Hospital and signed informed consent by parents and teens.

*Exclusion criteria:* BMI below $95^{th}$ percentile of local reference, concurrent participation in any clinical trial, dietary intervention or weight loss program, concomitant intake of a weight reducing agent, any medical illness or condition including known non-compliance, as judged by the investigators as ineligible to participate the study, unwillingness to attend regular follow up appointments as part of the intervention programme.

*Sample size calculation:* For this pilot study there will be 16 subjects in the control and 16 subjects in each of the two different intervention groups. There will be range of ages and both male and female participants.

*Ethics:* Written informed consent/assent will be obtained from teen subjects and their parents.

*Details: Focus groups and curriculum testing*: For the first five months of the study period, the internet curriculum will be developed, tested and refined through cognitive interviews involving approximately 6-8 adolescent patients ages 14-18 from the obesity clinic. *Recruitment and data collection*: From months five to six eligible subjects will be invited to participate in the pilot study. After obtaining informed consent (Appendix IIa-b), suitable subjects will be randomly assigned to either one of two intervention groups or control group using random numbers generated from computer (Appendix III). The control group will also receive usual care which consists of visits to the obesity clinic staffed by a paediatrician every three months while the first intervention group- will receive visits to the obesity clinic every three months plus do2 week internet curriculum and cell phone follow up over 6 months. The second intervention group will receive visits to the obesity clinic every three months and three meetings with a nutritionists (LMP)over three months .

All subjects will complete questionnaires regarding their physical activity, stress level and dietary intake at baseline (T0), 3 months (T12) and 6 months (T24) after the baseline assessment (Appendix IV ,V, IX))
The parents will also complete questionnaires regarding demographic information. (Appendix VI).
Subjects will be given $50 (book coupon) for transportation at week baseline, week 12 and week 24.
In addition to visits to the provider every three months, the first intervention group subjects will receive a web-based curriculum modelled on the ABC curriculum from weeks 0-12.
The internet curriculum (Appendix VII) will consist of twelve 15 minute interactive sessions using an articulated presentations developed by the Offices of Educational Services at The Chinese University of Hong Kong for student teaching. Material covered will include nutrition, exercise, and stress management based on the ABC curriculum. As in the ABC study, adolescents will be taught to set realistic goals regarding issues they are ready to make a change in and will be given the skills to enhance self-efficacy. Information related to nutrition such as the Food Pyramid and Portion Size and increasing energy expenditure through physical activity such as brisk walking and decreasing computer use will be discussed.

Subjects and parents will be asked to log in to the system with to participate in the sessions. Several questions will be asked at the end of the session to assess participant understanding and personal goals. Parents will also be asked to read the curriculum and complete online surveys regarding their teen's goals and progress. Participants and parents will also be allowed to add any personal comments regarding challenges and successes with diet and exercise. The comments will be reviewed by the research assistant and used for email and cell phone follow up and feedback. The RA will also discuss barriers to lifestyle change and child's and parents' feelings about the progress weekly. Intervention participants will be invited to join an online social media site using pseudonyms to discuss their participation in the program. The site will be moderated by the research assistant At the end of the program, parents and teens will also be asked for verbal and written comments regarding their satisfaction with the curriculum, nutritional counselling and telephone follow up.

In addition to visits to the provider every three months, the second intervention group subjects will come for nutritional counselling through the existing CUHK LMP centre at 2 weeks, 4 weeks and 12 weeks. During the initial hour-long assessment, the parent-teen pair and dietician will carry out a complete behavioural assessment and collect the medical history , current eating and lifestyle patterns and readiness to change. The dietician will also discuss specific dietary and lifestyle advice. During the follow up counselling of 15 to 20 minutes, the dietician will review a food record to check compliance on diet, exercise adherence and progression. A varied balanced diet with an emphasis on the American Dietetic Association guidelines with low-fat, low-glycaemic index and low calorie products in appropriate portions will be encouraged.

*Anthropometric assessments:* Anthropometric measurements, will be performed at baseline, 12 weeks and 24 weeks.Standing height without shoes will be measured using a Harpenden Stadiometer to the nearest 0.1 cm. Body weight will be measured using a portable Tanita scale (Model BF-522). Blood pressure will be measured using a Datascope Accutorr Plus.

*Diet and physical activity assessment:* Nutrient intake and consumption of food group of all subjects at baseline, 12 weeks and 24 weeks will be assessed using a 3-day dietary record and will be calculated by the nutrition analysis software Food Processor Nutrition analysis and Fitness software version 7.9 (Esna Research, Salem, USA) including local foods selected from food composition tables from China and Hong Kong. Subjects will also be asked to keep a weekly physical activity log, which they will submit by internet. In addition validated assessments regarding food group intake(12) and physical activity will be completed by all subjects at baseline, 12 and 24 weeks.

*Knowledge assessment*: Knowledge of dietary choices will be assessed at baseline, 12 and 24 weeks (13) (Appendix VIII).

Stress level: Stress level will be assessed at baseline, 12 weeks and 24 weeks using the 21-item standardized Chinese version of Depression, Anxiety and Stress Scale. (See appendix IX)

*Data analysis*: Descriptive statistics will be calculated for demographic characteristics, recruitment and retention rate. Average log-in rate and compliance with curriculum rate will be computed as a percentage of sessions the subject logs on and completes activities over 12 sessions. Compliance with curriculum and cell phone communication rates will also be computed as percentage. Data will be presented as mean (standard deviation), median (inter-quartile range) for skewed variables and frequency (percentage) as appropriate. Linear mixed effects models that include function of time and group effects and time-group interaction in repeated data will be used to examine the change in the data times between groups. Satisfaction scores with counselling, curriculum and cell phone communication will be presented as non-parametric paired comparisons using the Wilcoxon signed rank test. The level of physical activity level from baseline to follow up between groups, dietary intake, and knowledge related to nutrition as well as, weight and blood pressure will be expressed using multilevel models (a.k.a. linear mixed models) for longitudinal data. A p-value $< 0.05$ will be considered statistically significant. SPSS for Windows software (version 17.0, SPSS Inc., Chicago, IL, USA) will be used for the statistics.

**Appendix I: Effectiveness of the lifestyle modification programme (LMP)**

The LMP is an evidence-based method developed by the Centre for Nutritional Studies based on motivational interviewing and behavioural modification, to accompany improving knowledge regarding diet and exercise. The magnitude of weight reduction using this method is in the same order of magnitude as that achieved by pharmacological methods (Figure 1), but with an average of less than 10% weight re-bound at 12 months in a cohort of subjects (Table 1), and good compliance of over 70%.

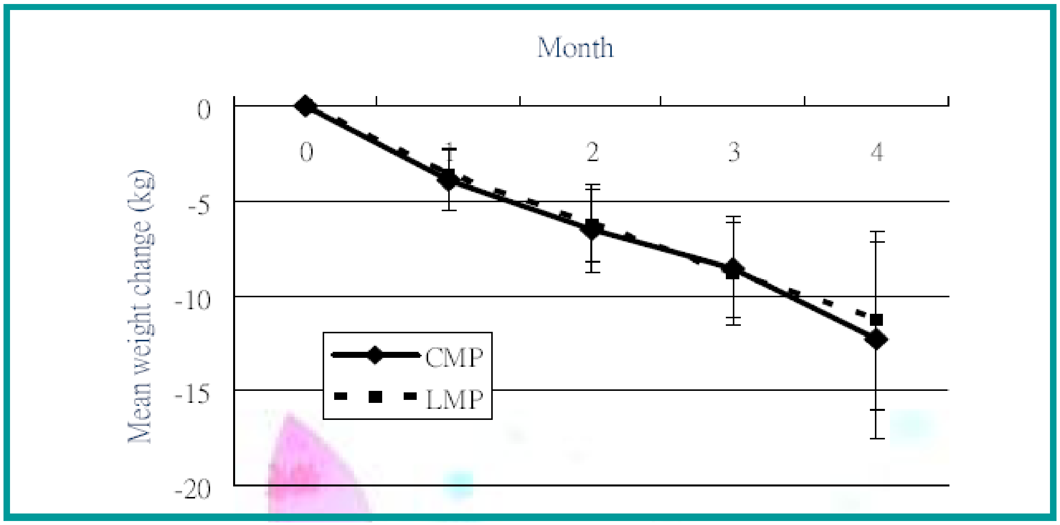


Figure 1 Mean weight change for matched subjects in comprehensive weight management programme with weight reducing drugs (CMP) and LMP

Table 1 Weight maintaining ability of CMP and LMP after 6 months of the programme

| | CMP (n=30) | LMP (n=63) |
|---|---|---|
| No. of subjects with weight rebound ≥ 2kg, n(%) | 5 (16.7%) | 6 (9.5%) |
| No. of subjects with weight rebound < 2kg, n(%) | 5 (16.7%) | 13 (19%) |
| No. of subjects with weight maintained or further reduced, n(%) | 20[a] (66.7%) | 44 (69.8%) |

[a]14 patients continued to take weight loss drug

**Appendix IIa:Consent Form-English version**

**Study title:** Lifestyle intervention using an internet-based curriculum with cell phone reminders among obese Chinese adolescents: A randomized controlled pilot study

**Researchers:** Edmund Anthony NELSON, Anisha ABRAHAM, Albert M. LI, Tan CHOW, Jean WOO, Ruth Suk Mei CHAN, Esther LAU and Hung Kwan SO

**Contact:** Dr. Hung Kwan SO - 2632 2829

**Background:** Obesity is an increasing public health problem affecting young people. Obesity is associated with a variety of physical and mental health problems including high blood pressure, diabetes, sleep disorders, and low-self-esteem. A web-based behavioural program with telephone follow up for teens and parents is an innovative means of improving traditional care. Results of this pilot study could inform a larger study and could lead to a more economical and creative means of changing weight and promoting a healthy lifestyle among teens.

**Aim:** To determine the feasibility of using an internet obesity lifestyle modification curriculum and nutritional counselling program along with cell phone follow up for teens.

**Nature of research**: This is a randomized controlled study with three possible study groups and you will be randomly allocated to one of these. This means that it is like a "flip of the coin" and that you cannot select which group you prefer to be part of. Once selected, you will be informed whether you are in the Intervention or Control group. The Control subjects will receive usual care in the obesity clinic which consists of follow up every three to six months. The first Intervention subjects will receive visits to the obesity clinic every three months plus do 12 week internet curriculum and cell phone follow up over 6 months. The first Intervention subjects and their parents will be given passwords to log onto a secure site to view online tutorials and will be able to answer questions and submit comments regarding the weight management program. The second Intervention subjects will receive visits to the obesity clinic every three months and have three sessions of nutritional counselling. All participants will be asked to complete surveys on nutritional intake, knowledge and confidence in diet and physical activity.

**Harms:** The investigations will not lead to any harm to the participants.

**Benefits:** Adolescents who take part in the study and are in the intervention group will be given a free of charge lifestyle intervention programme through nutritional counselling and an internet curriculum. The use of nutritional counselling with a web-based program could help participants to successfully improve their diet and increase physical activity.

**Confidentiality:** All participants' data will be protected by patient confidentiality. Information will be used for research purposes and will be accessed only by the investigators and research staff.

**Rights:** This study is compliant with the Declaration of Helsinki. Participation in research is entirely voluntary. Each participant has the right to refuse and the right to withdraw from the study without prejudice.

**Informed Consent Parent or Legally Authorized Representative:**
By signing this form below I confirm that this consent form has been explain to me in terms which I

understand. I consent my child_______________________, HKID___________________ to join the above study.

______________________________________________ ________________
Signature of Parent or Legally Authorized Representative Date

Name &Signature of Witness (if representative) Date

**Informed Assent by Participant:**
By signing this form below I confirm that this consent form has been explain to me in terms which I understand.

I assent to join the above study.

Signature of Participant Date

**Name & Signature of Investigator Date**

**Consent Form (IIa) (Chinese)**

**香港中文大學醫學院兒科學系**

**知情同意書**

研究負責人： 倪以信教授，Anisha ABRAHAM 教授，李民瞻教授，周馥丹女士，胡令芳教授，陳淑薇博士，劉月瑩教授，蘇鴻鈞博士

研究題目： 通過手機向肥胖青少年作提示互聯網生活方式干預方案的先導研究

背景：青少年肥胖是上升中的公共健康問題。肥胖與多種生理和心理健康問題相關聯，包括高血壓，代謝綜合症，睡眠障礙，抑鬱症和低自尊。針對以上問題，互聯網行為方案是青少年和家長的一個創新處理方法。香港從沒有使用互聯網進行肥胖青少年和家長的預防方案的相關報導，這先導研究的結果可帶領出和確定互聯網生活方式干預綜合方案對青少年體重和促進健康生活方式的效果。

目的：旨在確定通過手機提示使用互聯網生活方式干預綜合方案，青少年遵循這方案和營養諮詢的可行性。

內容：這是一個有三個研究小組的隨機對照研究，你會被隨機分配到其中的一個。就是說像擲硬幣的邏輯，你不能選擇你屬於哪一組。被隨機選定後，參與者將被分為三組，分別為研究組(1)、研究組(2)和對照組。對照組將按常規處理，包括每 3 個月 1 次的肥胖專科跟進，而研究組(1)將獲得 12 周的互聯網生活方式干預綜合方案的課程、每 3 個月 1 次的肥胖專科跟進和每週手機跟進提示。研究組(2)將會接受每 3 個月 1 次的肥胖專科跟進和 3 次營養諮詢。所有參與者將被要求填寫飲食營養及體育活動的相關知識和記錄。

弊處：本研究的所有項目都不會對參與者造成任何損害。

好處：參與者將在隨訪中可通過健康飲食和身體活動增加改善肥胖情況的機會。所有參與者都會獲得個人結果報告與家人分享。研究組的青少年將通過互聯網課程免費獲得營養諮詢和生活方式干預計劃的內容。

檔案保密：閣下所有資料均絕對保密，並只作研究用途。

權利：這研究嚴遵赫爾辛基宣言(Declaration of Helsinki)。你的參加是自願性質。你絕對有權拒絕參加這項研究或你有權在任何階段退出。
有關這項研究的問題，可聯絡蘇鴻鈞博士，電話：2632 2829。

**同意自願參加:**

本人____________________為__________________之家長／監護人，經研究人員詳細解釋後，同意參與以上之研究。對此研究之性質、目的及程序亦已經完全明白。

____________________________ ____________________________
參與者簽署 日期

____________________________ ____________________________
家長／監護人簽署 日期

____________________________ ____________________________
見證人姓名及簽署 日期

____________________________ ____________________________
研究員姓名及簽署 日期

## Appendix IIb:Consent Form-English versionfor the focus group

**Study title:** Lifestyle intervention using an internet-based curriculum with cell phone reminders among obese Chinese adolescents: A randomized controlled pilot study

**Researchers:** Edmund Anthony NELSON, Anisha AMRAHAM, Albert M. LI, Shekar KUMTA, Tan CHOW, Jean WOO, Ruth Suk Mei CHAN, Esther LAU and Hung Kwan SO
Contact: Miss Stella Chow-6755 8339, stella@cuhk.edu.hk

**Background:** Obesity is an increasing public health problem affecting young people. Obesity is associated with a variety of physical and mental health problems including high blood pressure, diabetes, sleep disorders, and low self-esteem. A web-based behavioral program with telephone follow up for teens and parents is an innovative means of improving traditional care. Results of this pilot study could inform a larger study and could lead to a more economical and creative means of changing weight and promoting a healthy lifestyle among teens.

**Aim:** To determine the feasibility of using an internet obesity lifestyle modification along with cell phone follow up for teens.

**Description of your involvement:** If you agree to be part of the research study, you will be asked participate in one focus group session. We will invite 6 to 8 people to meet together to provide feedback on the 12-week curriculum and the use of social media and texting in the study. The focus group will last about one-two hours and we will videotape the discussion to make sure that it is recorded accurately.

**Harms:** The investigations will not lead to any harm to the participants
Compensation: You will take a look at the 12-week internet curriculum and be paid HKD 50 book coupon for participating in the entire focus group session.
Confidentiality: All participants' data will be protected by patient confidentiality. Audiotaped conversations will be transcribed verbatim. Information will be used for research purposes and will be accessed only by the investigators and research staff.
**Rights:** This study is compliant with the Declaration of Helsinki. Participation in research is entirely voluntary. Each participant has the right to refuse and the right to withdraw from the study without prejudice.

**Informed Consent Parent or Legally Authorized Representative:**
By signing this form below I confirm that this consent form has been explained to me in terms which I

understand. I consent my child ______________________________, HKID______________ to join the above study.

______________________________ ______________________
Signature of Parent or Legally Authorized Representative Date

______________________________ ______________________
Signature of Witness (if representative) Date

**Informed Assent by Participant:**
By signing this form below I confirm that this consent form has been explained to me in terms which I understand.

I_____________________ assent to join the above study.

______________________________ ____________________
Signature of Participant Date

______________________________________________ ___________________
**Name & Signature of Investigator** **Date**

**Consent Form (IIb) (Chinese)**

## 香港中文大學醫學院兒科學系

## 知情同意書

研究負責人：倪以信教授，Anisha ABRAHAM 教授，李民瞻教授，周馥丹女士,胡令芳教授，陳淑薇博士，劉月瑩教授，蘇鴻鈞博士

研究題目：設計與評估肥胖青少年對互聯網生活方式干預綜合方案課程的可行性

背景：青少年肥胖是上升中的公共健康問題。肥胖與多種生理和心理健康問題相關聯，包括高血壓，代謝綜合症，睡眠障礙，抑鬱症和低自尊。針對以上問題，互聯網行為方案是青少年和家長的一個創新處理方法。香港從沒有使用互聯網進行肥胖青少年和家長的預防方案的相關報導，這聚焦小組的結果可帶領出和確定互聯網生活方式干預綜合方案對青少年體重和促進健康生活方式的可行性。

目的：旨在確定通過手機提示使用互聯網生活方式干預綜合方案，青少年遵循這方案的認受性及可行性。

內容：你會被要求參與是次聚焦小組。這次聚焦小組會有訪問員帶領 6 至 8 位被訪者一起就互聯網生活方式干預綜合方案 12 周課程的可行性以及手機提示深入討論以及發表意見。聚焦小組大約需要一至兩個小時的時間。為獲得準確的數據作分析，整個聚焦小組內容將會被錄音。

弊處：本研究的所有項目都不會對參與者造成任何損害。

好處：參與者如能成功完成是次聚焦小組，將會獲得價值港幣 50 元的書店禮卷並且可以瀏覽互聯網 12 周課程的內容。

檔案保密：閣下所有資料均絕對保密，並只作研究用途。錄音將被轉錄為文字並且保存在一個受密碼保護而只有參與本研究的人方可進入的電腦文件。

權利：這研究嚴遵赫爾辛基宣言(Declaration of Helsinki)。你的參加是自願性質。你絕對有權拒絕參加這項研究或你有權在任何階段退出。

有關這項研究的問題，可聯絡周泳芝小姐，電話：6755 8339 或
電子郵件:stella@cuhk.edu.hk

同意自願參加:

本人____________________為__________________之家長 / 監護人，經研究人員詳細解釋後，同意參與以上之研究。對此研究之性質、目的及程序亦已經完全明白。

___________________________ _______________
參與者簽署 日期

___________________________ _______________
家長 / 監護人簽署 日期

___________________________ _______________
見證人姓名及簽署 日期

___________________________ _________________
研究員姓名及簽署 日期

**Appendix III: Flow chart**

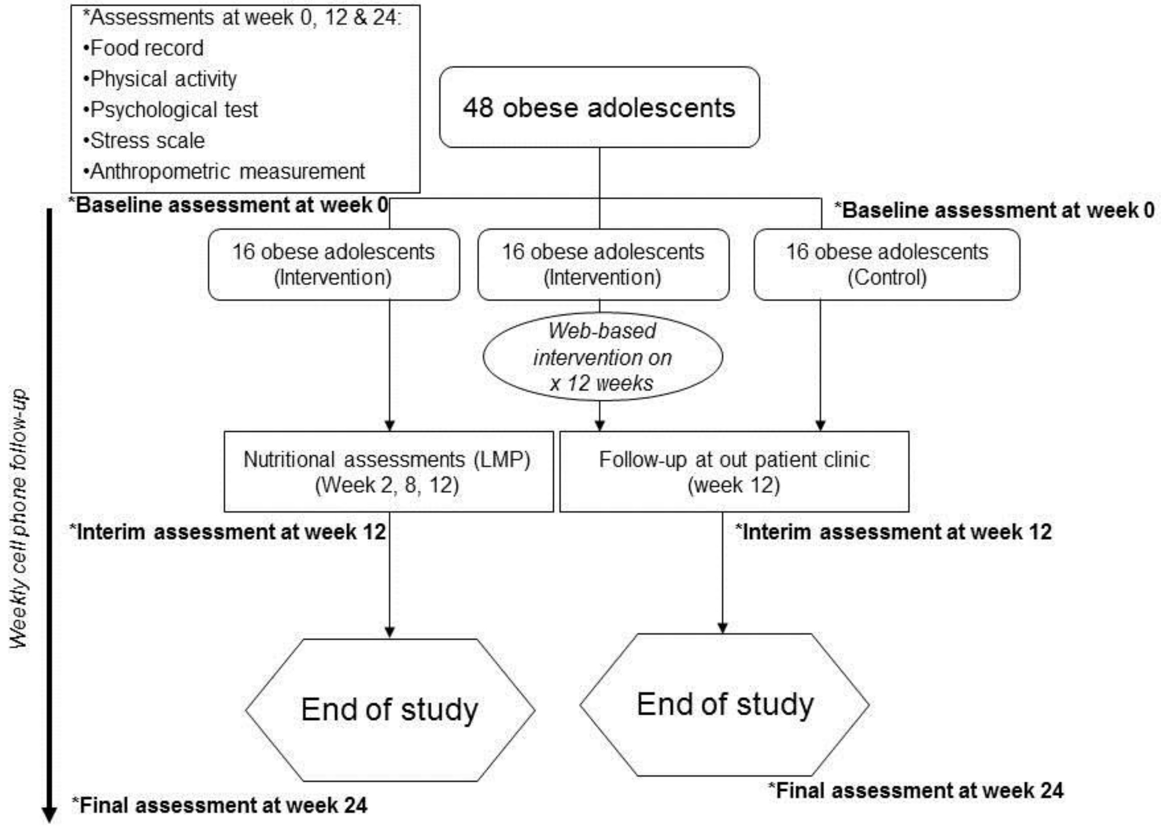

## Appendix IV: Physical Activity Assessment

CUHK Leisure Time Physical Activity Ratings for Children & Youth

From a scale of 0 to 10 listed below, that best describe your overall weekly level of physical activity in the past **12 MONTHS** period, then put the number in the square provided below: (refer to the annex table below for descriptions of light, moderate, and vigorous activities)

Select **ONLY ONE** rating and put into this square: → ☐

**Choose 0 to 2 if you have no exercise habits at all:**

**0** — no physical activity at all, spend most of your time sitting or sleeping.

**1** — no physical activity except little physical activity during PE lessons.

**2** — no physical activity except being active during PE lessons。

**Choose 3 to 6 if you, in addition to activity during PE lessons, participate in other physical activity occasionally:**

**3** — besides of the P.E. classes, I participate in light activities (3METs)# last longer than 20 minutes only once or twice every week.

**4** — besides of the P.E. classes, I participate in light activities last longer than 20 minutes for three times a week.

**5** — besides of the P.E. classes, I participate in light activities last longer than 20 minutes almost everyday.

**6** — besides of the P.E. classes, I participate in moderate activities (5METs) last longer than 20 minutes once or twice a week.

**Choose 7 to 10 if you, in addition to activity during PE lessons, participate in other physical activity regularly:**

**7** — besides of the P.E. classes, I participate in moderate activities last longer than 20 minutes three to five times a week.

**8** — besides of the P.E. classes, I participate in moderate activities last longer than 20 minutes almost everyday.

**9** — besides of the P.E. classes, I participate in vigorous activities (9METs) last longer than 20 minutes three times or less weekly.

**10**— besides of the P.E. classes, I participate in vigorous activities last longer than 20 minutes almost everyday.

*** Annex Table：Examples of Light, Moderate & Vigorous Activities**

| | **Light *(3METs)** | **Moderate *(5METs)** | **Vigorous *(9METs)** |
|---|---|---|---|
| **Home Activities** | Walk around home<br>Handle household appliances | Walk upstairs while lifting a 1-15 lbs weight | Moving large furniture upstairs |
| | Walk downstairs | Walk stairs up and down | |
| | Light playing while standing | Home exercise such as calisthenics, sit-up etc. | Vigorous activities such as push up |
| | Household cleaning such as mopping floor and vacuuming | Floor cleaning by hand; window or car washing which requires heavy physical exertion. | Carrying heavy groceries upstairs |
| **Sports Activities** | | Game playing inside water | Lap swimming |
| | Playing bowling | Basketball shooting (continuous) | Vigorous basketball competition |
| | Volleyball playing (1 ball for 6 – 9 persons) | Badminton playing (single or double) | Vigorous soccer competition |
| | Archery | Cycling; mixture of slow jogging and fast walking | Fast running (5.2mph or11.5min/mile) |
| | Frisbee | Playground playing | Orienteering |
| | Dancing in a dance room (slow dance, waltz) | Low impact aerobic dance & folk dance | Cross-country running / skiing |
| **Others** | Guitar playing (standing) | Band marching | |

**# METs is Metabolic Equivalent, 1 MET is the energy expenditure during rest，3METs is three times the energy expenditure of resting，6METs is six times, and so on.**

**中大學童課餘運動量評級問卷**

請您於下列 0-10 的等級中，選取**其中一等級**代表您過去一年內**平均每星期**的運動量，然後填入下面方格中：**(請參考下列附表*中有關低強度、中等強度、及劇烈運動的例子)**

從下表 0-10 的等級中，**祇選一等級**填入此方格 → ☐

**沒有運動習慣者，選 0 至 2**

**0**—完全沒有任何運動，大部份時間是坐著或睡覺。
**1**—除了在體育課有少許活動外，其餘所有時間都沒有運動。
**2**—除了上體育課時有積極參與運動外，其餘所有時間都沒有運動。

**除了上體育課有運動外，平時間中有運動習慣者，選 3 至 6**

**3**—每星期都有一至兩次 20 分鐘以上低強度運動*（**3 METs**#）。
**4**—每星期都有三次以上 20 分鐘以上低強度運動。
**5**—差不多每天都有一次 20 分鐘以上低強度運動。
**6**—每星期都有一至兩次 20 分鐘以上中等強度運動*（**5 METs**）。

**除了上體育課有運動外，平時經常有運動習慣者，選 7 至 10**

**7**—每星期都有三次至五次中等強度運動(每次 20 分鐘或以上)。
**8**—差不多每天都有中等強度運動(每次 20 分鐘或以上)。
**9**—每星期都有不多於三次劇烈運動*（**9 METs**）(每次 20 分鐘或以上)。
**10**—差不多每天都有劇烈運動(每次 20 分鐘或以上)。

*** 附表：低強度、中等強度、及劇烈運動的例子**

| | **低強度運動 *(3METs)** | **中等運動 *(5METs)** | **劇烈運動 *(9METs)** |
|---|---|---|---|
| **家居活動** | 在家中行走及搬動家居用品 | 提取 1-15 磅的物品步行上樓梯 | 將家俬或大型家居搬運上樓梯 |
| | 落樓梯 | 上落樓梯混合 | |
| | 在家中站立而輕量的玩耍 | 家中的運動，如柔軟體操、仰臥起坐等 | 劇烈的運動(很辛苦的掌上壓) |
| | 做家務，如抹地吸塵或清潔家居 | 用手或跪地型式的抹地；較辛苦的家務，如抹窗或洗車 | |
| **體育運動** | | 嬉戲型式的游泳 | 不斷來回型式的游泳 |
| | 保齡球 | 籃球練習(如不斷射籃) | 劇烈的籃球比賽 |
| | 排球對打(6-9 人使用一個排球) | 羽毛球對打練習 (單人或雙人) | 劇烈的足球比賽 |
| | 射箭 | 踏單車；混合慢跑及步行 | 比一般跑步快一些（幾吃力）；即以 5.2mph(11.5min/mile)的速度跑步 |
| | 玩飛碟 | 於遊樂場遊玩 | 越野跑步 |
| | 於舞蹈室內的舞蹈(如慢舞或華爾茲) | 舞蹈(低衝擊性的健康舞、體育課的土風舞) | 野外定向 |
| **其他** | 結他(站立) | | |
| | 管弦樂隊(步操) | | |

**# METs 是能量等值單位，1 MET 是靜止時能量消耗，3METs 即能量消耗為靜止時的 3 倍，6METs 是六倍，餘類推。**

**Appendix V: Dietary Assessment**

| | **Monthly** | | **Weekly** | | | **Daily** | | | **Don’t know** |
|---|---|---|---|---|---|---|---|---|---|
| | **<1 time/ don’t eat** | **1-3 time (s)** | **Once** | **2-4 times** | **5-6 times** | **Once** | **Twice** | **>Twice** | |
| 1. Fruit | □$_1$ | □$_2$ | □$_3$ | □$_4$ | □$_5$ | □$_6$ | □$_7$ | □$_8$ | □$_9$ |
| How many fruits do you eat a day? *(one fruit equals to one apple, orange, pear, etc) ________ | | | | | | | | | |
| 2. Vegetable | □$_1$ | □$_2$ | □$_3$ | □$_4$ | □$_5$ | □$_6$ | □$_7$ | □$_8$ | □$_9$ |
| How many bowls of vegetable do you eat a day? *(one bowl refers to size of rice bowl) ________ | | | | | | | | | |
| 3. Soybean curd | □$_1$ | □$_2$ | □$_3$ | □$_4$ | □$_5$ | □$_6$ | □$_7$ | □$_8$ | □$_9$ |
| 4. Milk/milk product | □$_1$ | □$_2$ | □$_3$ | □$_4$ | □$_5$ | □$_6$ | □$_7$ | □$_8$ | □$_9$ |
| 5. Chinese tea (e.g. green tea) | □$_1$ | □$_2$ | □$_3$ | □$_4$ | □$_5$ | □$_6$ | □$_7$ | □$_8$ | □$_9$ |
| 6. Fish (excluding salted fish) | □$_1$ | □$_2$ | □$_3$ | □$_4$ | □$_5$ | □$_6$ | □$_7$ | □$_8$ | □$_9$ |
| 7. Meat (e.g. pork, beef, poultry) | □$_1$ | □$_2$ | □$_3$ | □$_4$ | □$_5$ | □$_6$ | □$_7$ | □$_8$ | □$_9$ |
| 8. Deep-fried and high fat food | □$_1$ | □$_2$ | □$_3$ | □$_4$ | □$_5$ | □$_6$ | □$_7$ | □$_8$ | □$_9$ |

## 【健康評估及調查】

編號: ________________ 訪問日期: ________________

**食物頻率問卷**

| | **每月** | | **每星期** | | | **每日** | | | **唔知道** |
|---|---|---|---|---|---|---|---|---|---|
| | **<1 次/唔食** | **1-3 次** | **1 次** | **2-4 次** | **5-6 次** | **1 次** | **2 次** | **>2 次** | |
| 1. 你進食新鮮水果平均有幾頻密? | $\square_1$ | $\square_2$ | $\square_3$ | $\square_4$ | $\square_5$ | $\square_6$ | $\square_7$ | $\square_8$ | $\square_9$ |
| 2. 咁你每日總共食幾多個水果? *(一個水果大約等於一個蘋果,橙,梨) ____個 | | | | | | | | | |
| 3. 新鮮蔬菜? | $\square_1$ | $\square_2$ | $\square_3$ | $\square_4$ | $\square_5$ | $\square_6$ | $\square_7$ | $\square_8$ | $\square_9$ |
| 4. 咁你每日總共食幾多碗菜? *(以普通飯碗計) ____碗 | | | | | | | | | |
| 5. 豆腐? | $\square_1$ | $\square_2$ | $\square_3$ | $\square_4$ | $\square_5$ | $\square_6$ | $\square_7$ | $\square_8$ | $\square_9$ |
| 6. 牛奶/奶品類? | $\square_1$ | $\square_2$ | $\square_3$ | $\square_4$ | $\square_5$ | $\square_6$ | $\square_7$ | $\square_8$ | $\square_9$ |
| 7. 中國茶 (包括綠茶)? | $\square_1$ | $\square_2$ | $\square_3$ | $\square_4$ | $\square_5$ | $\square_6$ | $\square_7$ | $\square_8$ | $\square_9$ |
| 8. 魚類 (不包括鹹魚)? | $\square_1$ | $\square_2$ | $\square_3$ | $\square_4$ | $\square_5$ | $\square_6$ | $\square_7$ | $\square_8$ | $\square_9$ |
| 9. 其他肉類 (如豬、牛、家禽)? | $\square_1$ | $\square_2$ | $\square_3$ | $\square_4$ | $\square_5$ | $\square_6$ | $\square_7$ | $\square_8$ | $\square_9$ |
| 10. 煎炸、含高脂肪嘅食物? | $\square_1$ | $\square_2$ | $\square_3$ | $\square_4$ | $\square_5$ | $\square_6$ | $\square_7$ | $\square_8$ | $\square_9$ |

**Appendix VI: Demographic information**

**Patient no.:**

**Birth weight:**kg

**Gestational age:**□ full term (>= 37 wks) □ preterm wks

**Infant feeding:**□ Never Breastfed □ Breastfed < 4 weeks □ Breastfed < 3 months

□ Breastfed > 3 months

**Father's education level:** □ 1.primary □ 2. secondary□ 3. post-secondary□ 4. tertiary or above

**Mother's: education level:** □ 1. Primary □ 2. secondary□ 3. post-secondary□ 4. tertiary or above

**Mother's height:** cm or feet inch

**Father's height:** cm or feet inch

**Mother's weight:** lb or kg

**Father's weight:** lb or kg

**Family History in immediate family:** please tick if following diseases present in child's mother or father or brother or sister: □ 1.High blood pressure □ 2.Stroke □ 3.Coronary heart disease

□4.Diabetes □ 5.Obesity

**Adolescent's sleep routine:** Usual bedtime hrmin Time awakehrmin

**Physical activity:** Do you have any regular sport activity training besides of PE? □1.Yes □ 2.no

What is the frequency of training: □ 1.once/week □ 2.twice/week □3.three times or more/week

家庭資料

**參加者編號：**

**出生時體重：**磅　　　/ _公斤

**出生時之懷孕周數：**□1.足月（> = 37 周）　　　□2.早產（懷孕周數）周

**嬰孩時哺育：**□1.從未母乳哺育　　　□2.母乳哺育< 4 星期

□3.母乳哺育< 3 個月　　　□4.母乳哺育> 3 個月

**父親教育程度：**　　□1. 小學或以下　□2. 中學程度　□3.中學畢業　□4.大專或以上

**母親教育程度：**　　□1. 小學或以下　□2. 中學程度　□3.中學畢業　□4.大專或以上

**父親的身高：** cm　　　/ 呎吋

**母親的身高：**cm　　　/ 呎吋

**父親的體重：**磅　　　/ 公斤

**母親的體重：**磅　　　/ 公斤

**現時家庭病歷資料：**如父母或兄弟姊妹成員有以下的病歷，請在□內加√

□1.高血壓　　□2.中風　　□3.冠心病　　□4.糖尿病　　□5.肥胖症

**兒童睡眠常態：**上床時間時分　　　起床時分

除體育課外你每週有否接受其它定期的體育訓練？□1.有　　□2.沒有

每週訓練次數：□1.一次　　□2.兩次　　□3.三次或以上

(以上調查資料只作統計用途，並且絕對保密)

**Appendix VII: Internet Curriculum**

| Lesson | Intervention |
|---|---|
| 1 | Introduction-Basic definitions and welcome to internet curriculum |
| 2 | Obesity Causes and Consequences-understand the main reasons for increasing weight and what effects this can have on your health |
| 3 | Nutrition Basics-Food Pyramid- understand food and health |
| 4 | Nutrition Basics- learn about portion size and how to make smart food choices |
| 5 | Eating healthy outside of the home |
| 6 | Physical Activity- Understand the importance of an adequate activity level |
| 7 | Physical Activity- Being cool and active: various fun activities for youth and families |
| 8 | Physical Activity- Being yourself and using fun ways to improve your health and maintain a healthy lifestyle |
| 9 | TV/computer time-Learn alternatives to watching TV and using the computer |
| 10 | Stress/coping- Understand how the body works and how to recognize and cope with feelings |
| 11 | Stress/coping- Use various relaxation techniques and develop healthy coping |
| 12 | Wrap-Up |

**Appendix VIII: Nutrition Knowledge**

Reference number: _______________ Date: _______________

1. Do you know about the Food Guide Pyramid? □$_0$ No □$_1$ Yes

**Diet knowledge**

| **Statement** <br><br> *** Please note that the question is not asking about your actual habits** | **Strongly disagree** | **Disagree** | **Neutral** | **Agree** | **Strongly agree** | **Unknown** |
|---|---|---|---|---|---|---|
| 2. Choosing a diet with a lot of fresh fruits and vegetable is good for one's health. | □$_1$ | □$_2$ | □$_3$ | □$_4$ | □$_5$ | □$_9$ |
| 3. Eating a lot of sugar is food for one's health. | □$_1$ | □$_2$ | □$_3$ | □$_4$ | □$_5$ | □$_9$ |
| 4. Eating a variety of foods is good for one's health. | □$_1$ | □$_2$ | □$_3$ | □$_4$ | □$_5$ | □$_9$ |
| 5. Choosing a diet high in fat is good one's health. | □$_1$ | □$_2$ | □$_3$ | □$_4$ | □$_5$ | □$_9$ |
| 6. Choosing a diet with a lot of staple foods (rice and rice products and wheat and wheat products) is not good for one's health. | □$_1$ | □$_2$ | □$_3$ | □$_4$ | □$_5$ | □$_9$ |
| 7. Consuming a lot of animal products daily (fish, poultry, eggs, and lean meat) is good for one's health. | □$_1$ | □$_2$ | □$_3$ | □$_4$ | □$_5$ | □$_9$ |
| 8. Reducing the amount of fatty meat and animal fat in the diet is food for one's health. | □$_1$ | □$_2$ | □$_3$ | □$_4$ | □$_5$ | □$_9$ |
| 9. Consuming milk and dairy products is good for one's health. | □$_1$ | □$_2$ | □$_3$ | □$_4$ | □$_5$ | □$_9$ |
| 10. Consuming beans and bean products is good for one's health. | □$_1$ | □$_2$ | □$_3$ | □$_4$ | □$_5$ | □$_9$ |
| 11. Physical activities are good for one's health. | □$_1$ | □$_2$ | □$_3$ | □$_4$ | □$_5$ | □$_9$ |
| 12. Sweaty sports or other intense physical activities are not good for one's health. | □$_1$ | □$_2$ | □$_3$ | □$_4$ | □$_5$ | □$_9$ |
| 13. The heavier one's body is, the healthier he or she is. | □$_1$ | □$_2$ | □$_3$ | □$_4$ | □$_5$ | □$_9$ |

**Food preferences**

| **Food Item** | **Dislike very much** | **Dislike** | **Neutral** | **Like** | **Like very much** | **Do not eat this food** |
|---|---|---|---|---|---|---|
| 14. Fast food (KFC, pizza, hamburgers, etc) | □$_1$ | □$_2$ | □$_3$ | □$_4$ | □$_5$ | □$_9$ |
| 15. Salty snack foods (potato chips, pretzels, French fries, etc) | □$_1$ | □$_2$ | □$_3$ | □$_4$ | □$_5$ | □$_9$ |
| 16. Fruits | □$_1$ | □$_2$ | □$_3$ | □$_4$ | □$_5$ | □$_9$ |
| 17. Vegetables | □$_1$ | □$_2$ | □$_3$ | □$_4$ | □$_5$ | □$_9$ |

| | | | | | | |
|---|---|---|---|---|---|---|
| 18. Soft drinks and sugared fruit drinks | □$_1$ | □$_2$ | □$_3$ | □$_4$ | □$_5$ | □$_9$ |

# 飲食知識

編號: _______________　　　　　　　　訪問日期: _______________

1. 你認識健康飲食金字塔嗎？　□$_0$ 不知道　　□$_1$ 知道

**膳食知識 --** 請用☑表示你認為最合適的答案。

| **觀點描述**<br>*** 注意，以下只是問你的觀點，不是詢問你的實際習慣。** | **很不贊同** | **不贊同** | **中立** | **贊同** | **很贊同** | **不知道** |
|---|---|---|---|---|---|---|
| 2. 吃很多水果和蔬菜的飲食習慣對健康非常有益。 | □$_1$ | □$_2$ | □$_3$ | □$_4$ | □$_5$ | □$_9$ |
| 3. 多吃糖對健康有益。 | □$_1$ | □$_2$ | □$_3$ | □$_4$ | □$_5$ | □$_9$ |
| 4. 吃不同種類的食物對健康有益。 | □$_1$ | □$_2$ | □$_3$ | □$_4$ | □$_5$ | □$_9$ |
| 5. 吃高脂肪的食物對健康有益。 | □$_1$ | □$_2$ | □$_3$ | □$_4$ | □$_5$ | □$_9$ |
| 6. 吃大量穀類的飲食習慣（如米、小麥）是不利於健康的。 | □$_1$ | □$_2$ | □$_3$ | □$_4$ | □$_5$ | □$_9$ |
| 7. 每天吃很多肉類食品（如魚、雞肉、豬肉）對健康有益。 | □$_1$ | □$_2$ | □$_3$ | □$_4$ | □$_5$ | □$_9$ |
| 8. 吃飯時少吃肥肉和動物脂肪對健康有益。 | □$_1$ | □$_2$ | □$_3$ | □$_4$ | □$_5$ | □$_9$ |
| 9. 喝奶和吃乳製品對健康有益。 | □$_1$ | □$_2$ | □$_3$ | □$_4$ | □$_5$ | □$_9$ |
| 10. 吃豆及豆製品對健康有益。 | □$_1$ | □$_2$ | □$_3$ | □$_4$ | □$_5$ | □$_9$ |
| 11. 運動對健康有益。 | □$_1$ | □$_2$ | □$_3$ | □$_4$ | □$_5$ | □$_9$ |
| 12. 大運動量和劇烈的活動都是不利於健康的。 | □$_1$ | □$_2$ | □$_3$ | □$_4$ | □$_5$ | □$_9$ |
| 13. 身體越重，就越健康。 | □$_1$ | □$_2$ | □$_3$ | □$_4$ | □$_5$ | □$_9$ |

**食品偏好 --** 請用☑表示你喜歡這些食品的程度如何。

| **食品名稱** | **很不喜歡** | **不太喜歡** | **中立** | **有些喜歡** | **很喜歡** | **不知道** |
|---|---|---|---|---|---|---|
| 14. 快餐(麥當勞，肯德基，薄餅，漢堡包等) | □$_1$ | □$_2$ | □$_3$ | □$_4$ | □$_5$ | □$_9$ |
| 15. 鹹的零食(薯片，餅乾，薯條等) | □$_1$ | □$_2$ | □$_3$ | □$_4$ | □$_5$ | □$_9$ |
| 16. 水果 | □$_1$ | □$_2$ | □$_3$ | □$_4$ | □$_5$ | □$_9$ |
| 17. 蔬菜 | □$_1$ | □$_2$ | □$_3$ | □$_4$ | □$_5$ | □$_9$ |

| | | | | | | | |
|---|---|---|---|---|---|---|---|
| 18. | 汽水及含糖的果汁飲料 | □1 | □2 | □3 | □4 | □5 | □9 |

**Appendix IX: Depression, Anxiety and Stress Scale; English version**

# DASS21

*Name:* *Date:*

Please read each statement and circle a number 0, 1, 2 or 3 which indicates how much the statement applied to you *over the past week*. There are no right or wrong answers. Do not spend too much time on any statement.

*The rating scale is as follows:*

0 Did not apply to me at all

1 Applied to me to some degree, or some of the time

2 Applied to me to a considerable degree, or a good part of time

3 Applied to me very much, or most of the time

| | | | | | |
|---|---|---|---|---|---|
| 1 | I found it hard to wind down | 0 | 1 | 2 | 3 |
| 2 | I was aware of dryness of my mouth | 0 | 1 | 2 | 3 |
| 3 | I couldn't seem to experience any positive feeling at all | 0 | 1 | 2 | 3 |
| 4 | I experienced breathing difficulty (eg, excessively rapid breathing, breathlessness in the absence of physical exertion) | 0 | 1 | 2 | 3 |
| 5 | I found it difficult to work up the initiative to do things | 0 | 1 | 2 | 3 |
| 6 | I tended to over-react to situations | 0 | 1 | 2 | 3 |
| 7 | I experienced trembling (eg, in the hands) | 0 | 1 | 2 | 3 |
| 8 | I felt that I was using a lot of nervous energy | 0 | 1 | 2 | 3 |
| 9 | I was worried about situations in which I might panic and make a fool of myself | 0 | 1 | 2 | 3 |
| 10 | I felt that I had nothing to look forward to | 0 | 1 | 2 | 3 |
| 11 | I found myself getting agitated | 0 | 1 | 2 | 3 |
| 12 | I found it difficult to relax | 0 | 1 | 2 | 3 |
| 13 | I felt down-hearted and blue | 0 | 1 | 2 | 3 |

| | | | | | |
|---|---|---|---|---|---|
| 14 | I was intolerant of anything that kept me from getting on with what I was doing | 0 | 1 | 2 | 3 |
| 15 | I felt I was close to panic | 0 | 1 | 2 | 3 |
| 16 | I was unable to become enthusiastic about anything | 0 | 1 | 2 | 3 |
| 17 | I felt I wasn't worth much as a person | 0 | 1 | 2 | 3 |
| 18 | I felt that I was rather touchy | 0 | 1 | 2 | 3 |
| 19 | I was aware of the action of my heart in the absence of physical exertion (eg, sense of heart rate increase, heart missing a beat) | 0 | 1 | 2 | 3 |
| 20 | I felt scared without any good reason | 0 | 1 | 2 | 3 |
| 21 | I felt that life was meaningless | 0 | 1 | 2 | 3 |

**Appendix IX: Depression, Anxiety and Stress Scale; Chinese version**

# DASS21 姓名： 日期：

填表說明：

請小心閱讀以下每一個句子，並在其右方圈上一數字，表示「過往一個星期」如何適用於你。答案並無對錯之分。請不要花太多時間在某一句子上。

評估量表：

0 = 不適用

1 = 頗適用，或間中適用

2 = 很適用，或經常適用

3 = 最適用，或常常適用

| | | | | | |
|---|---|---|---|---|---|
| 1 | 我覺得很難讓自己安靜下來 | 0 | 1 | 2 | 3 |
| 2 | 我感到口乾 | 0 | 1 | 2 | 3 |
| 3 | 我好像不能再有任何愉快、舒暢的感覺 | 0 | 1 | 2 | 3 |
| 4 | 我感到呼吸困難（例如不是做運動時也感到氣促或透不過氣來） | 0 | 1 | 2 | 3 |
| 5 | 我感到很難自動去開始工作 | 0 | 1 | 2 | 3 |
| 6 | 我對事情往往作出過敏反應 | 0 | 1 | 2 | 3 |
| 7 | 我感到顫抖（例如手震） | 0 | 1 | 2 | 3 |
| 8 | 我覺得自己消耗很多精神 | 0 | 1 | 2 | 3 |
| 9 | 我憂慮一些令自己恐慌或出醜的場合 | 0 | 1 | 2 | 3 |
| 10 | 我覺得自己對將來沒有甚麼可盼望 | 0 | 1 | 2 | 3 |
| 11 | 我感到忐忑不安 | 0 | 1 | 2 | 3 |
| 12 | 我感到很難放鬆自己 | 0 | 1 | 2 | 3 |
| 13 | 我感到憂鬱沮喪 | 0 | 1 | 2 | 3 |
| 14 | 我無法容忍任何阻礙我繼續工作的事情 | 0 | 1 | 2 | 3 |
| 15 | 我感到快要恐慌了 | 0 | 1 | 2 | 3 |
| 16 | 我對任何事也不能熱衷 | 0 | 1 | 2 | 3 |

| | | | | | |
|---|---|---|---|---|---|
| 17 | 我覺得自己不怎麼配做人 | 0 | 1 | 2 | 3 |
| 18 | 我發覺自己很容易被觸怒 | 0 | 1 | 2 | 3 |
| 19 | 我察覺自己在沒有明顯的體力勞動時，也感到心律不正常 | 0 | 1 | 2 | 3 |
| 20 | 我無緣無故地感到害怕 | 0 | 1 | 2 | 3 |
| 21 | 我感到生命毫無意義 | 0 | 1 | 2 | 3 |
